# Neuronal replacement therapy: previous achievements and challenges ahead

**DOI:** 10.1038/s41536-017-0033-0

**Published:** 2017-10-23

**Authors:** Sofia Grade, Magdalena Götz

**Affiliations:** 1 Physiological Genomics, Biomedical Center, Ludwig-Maximilians University Munich, 82152 Planegg-Martinsried, Germany; 2 Institute of Stem Cell Research, Helmholtz Center Munich, German Research Center for Environmental Health, 85764 Neuherberg, Germany; 3 SYNERGY, Excellence Cluster of Systems Neurology, Biomedical Center, Ludwig-Maximilians University Munich, 82152 Planegg-Martinsried, Germany

## Abstract

Lifelong neurogenesis and incorporation of newborn neurons into mature neuronal circuits operates in specialized niches of the mammalian brain and serves as role model for neuronal replacement strategies. However, to which extent can the remaining brain parenchyma, which never incorporates new neurons during the adulthood, be as plastic and readily accommodate neurons in networks that suffered neuronal loss due to injury or neurological disease? Which microenvironment is permissive for neuronal replacement and synaptic integration and which cells perform best? Can lost function be restored and how adequate is the participation in the pre-existing circuitry? Could aberrant connections cause malfunction especially in networks dominated by excitatory neurons, such as the cerebral cortex? These questions show how important connectivity and circuitry aspects are for regenerative medicine, which is the focus of this review. We will discuss the impressive advances in neuronal replacement strategies and success from exogenous as well as endogenous cell sources. Both have seen key novel technologies, like the groundbreaking discovery of induced pluripotent stem cells and direct neuronal reprogramming, offering alternatives to the transplantation of fetal neurons, and both herald great expectations. For these to become reality, neuronal circuitry analysis is key now. As our understanding of neuronal circuits increases, neuronal replacement therapy should fulfill those prerequisites in network structure and function, in brain-wide input and output. Now is the time to incorporate neural circuitry research into regenerative medicine if we ever want to truly repair brain injury.

## Introduction

Central nervous system (CNS) degeneration or damage lead to irreversible neuronal loss and often persistent functional deficits constituting highly debilitating pathologies associated with a significant health and economic burden for patients, families, and societies. The available treatments aim to rescue the remaining neurons and rely on supportive care to compensate lack of neurotransmitters or alleviate symptoms, and on rehabilitation to promote brain functional plasticity.

While the CNS of mammals and birds, as opposed to other vertebrates, by and large fails to regenerate, it does hold a certain capacity to react to and compensate for cell loss, be that neurons or glia. In pathologies associated with a primary neuronal loss, which will be the focus of this review, a substantial amount of network restructuring and synaptic plasticity takes place, reducing the functional impairments or even masking the disease. In line with this, Parkinson’s disease (PD) becomes symptomatic when almost 80% of the nigrostriatal dopaminergic innervation is lost.^1^ Curiously, functional imaging in people at genetic risk of Alzheimer’s disease (AD) revealed increased signal intensity in circuits recruited for a given memory task, as compared to controls, despite equal performance.^2^ The greater circuit activation, possibly by recruiting more neurons to fire, or augmenting the firing rate of the same neuronal population, suggests that the brain utilizes additional resources to maintain performance despite loss of some neurons. Most impressively, functional compensation can occur via mobilization of other brain regions and connections to serve the motor, sensory, or cognitive demand that was previously performed by the lost neurons. This is the case in stroke patients where rehabilitation and/or deep brain stimulation engage surviving networks to take over a lost function, by structural and functional changes in the individual’s connectome.^3^ Likewise, functional recovery after incomplete spinal cord injury (SCI) results from spontaneous axonal sprouting from spared circuitries^4,5^ and voluntary movement after complete hindlimb paralysis can be encouraged by combining a set of activity-based interventions.^6^ To some extent, CNS injury awakens mechanisms of plasticity that thrive during CNS development, a stage when perturbation of wiring networks triggers the most successful compensatory routes. For instance, dysgenesis of the corpus callosum in human brain development is compensated by sprouting of connections via ventral commissures that sustain normal interhemispheric transfer and explain the lack of disconnection syndrome described otherwise in callosotomized patients.^7^ In summary, the mammalian brain displays an inherent capacity for functional homeostasis, using compensatory mechanisms that counteract injury-induced or disease-induced changes in the connectome as an attempt to preserve adequate brain function.^8–10^ This plasticity is, however, limited, especially in cases of extensive injury or in progressive diseases in which the brain accumulates dysfunction and inflammation, and patients acquire permanent disabilities. These cases are subject of our review that discusses potential neuronal replacement strategies to restore function. We will focus on discussing neuronal replacement strategies for the brain, as therapeutic approaches for SCI focus predominantly on glial cell replacement and axonal regeneration (for recent review see Assinck et al.^11^).

**Fig. 1:**
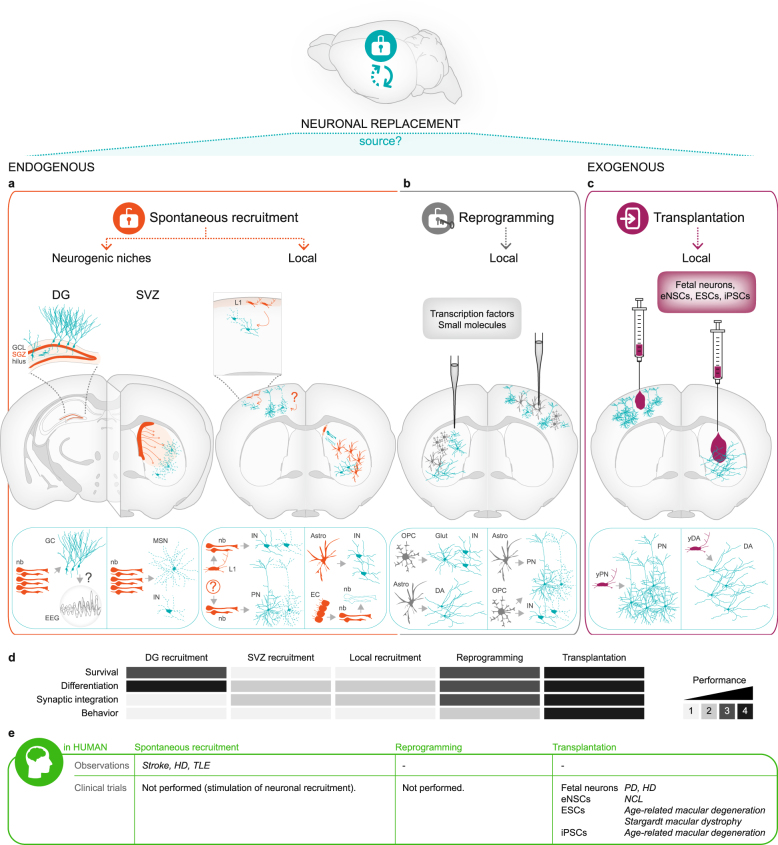
Neuronal replacement therapy for the brain. Overview of the approaches using endogenous (**a**, **b**) or exogenous cell sources (**c**). Neuron types and outcomes achieved are illustrated in coronal views of the rodent brain and highlighted on the schemes below. All examples depicted are based on studies in rodent models of brain injury or disease. Endogenous approaches include **a** recruitment from the brain neurogenic niches (DG or SVZ) or from latent local progenitors (cortical L1 or other; ependymal cells or striatal glia) (spontaneous, as illustrated by the opened locker; orange), and **b** neuronal reprogramming which converts local glial cells into neurons (forced, as illustrated by the locker opened by a key that symbolizes the reprogramming transcription factors/small molecules; gray). **c** Exogenous approaches (purple) use different sources of donor cells for transplantation into the injured or diseased area, including fetal neurons, eNSCs-derived, ESC-derived and iPSC-derived neurons. **a**–**c** Overall, neuronal replacement approaches have been performed mostly in the striatum or cortex. In the coronal views of the rodent brain, orange, gray, or purple refers to the region/cells of origin and blue refers to the neurons generated (note that the differentiation in glia, e.g., from SVZ or ependyma, is not illustrated). Among the latter, dashed lines represent cell death. Number of blue cells depicted in solid lines, the surviving neurons, informs of their relative survival. **d** Color-coded matrix represents the current and average outcome from each approach indicated on the top, in regard to the criteria indicated on the left, namely long-term survival, differentiation, and synaptic integration of the new neurons (blue in A–C schemes), as well as behavioral improvements. Grayscale: maximum success in the respective criterion is highlighted by the darkest gray (4) and failure to achieve any is shown in the lightest one (1). In case of synaptic integration the Grayscale means (1) integration is aberrant and perhaps detrimental, as in epilepsy, (2) only labeling for synaptic proteins and/or axonal projections to one target area was reported, (3) spontaneous postsynaptic currents were recorded and transsynaptic tracing with only local input was observed, and (4) full connectome (correct afferents and efferents) and functional properties as assessed by physiological measurements were achieved. **e** Data/clinical trials in patients. Only brain diseases or injuries of primary neuronal loss are shown here in agreement with the focus of the present review. *Abbreviations*: *Astro* astrocytes, *DA* *dopaminergic neurons*, *DG* *dentate gyrus*, *EC* *ependymal cells*, *EEG* *electroencephalogram*, *eNSCs* *embryonic neural stem cells*, *ESCs* *embryonic pluripotent stem cells*, *GC* *granule cells*, *GCL* granule cell layer, *Glut* *glutamatergic neurons*, *HD* Huntington’s disease, *IN* interneurons, *iPSCs* induced pluripotent stem cells, *L1* neocortex layer 1, *MSN* medium spiny neurons, *nb* neuroblasts, *NCLs* neuronal ceroid lipofuscinoses, *OPC* oligodendrocyte progenitor cells, *PD* Parkinson’s disease, *PN* projection neurons, *SGZ* subgranular zone, *SVZ* subventricular zone, *TLE* temporal lobe epilepsy, *yDA* young dopaminergic neurons, *yPN* young projection neurons

At first sight, substitution of a dying neuron by a new one within an extremely complex and intricate meshwork of connections, which are finely tuned during development sounds like a daunting challenge. However, the landmark discovery that also the adult mammalian brain shelters neural stem cells (NSCs) that continuously generate newborn neurons integrating into pre-existing neuronal circuitries substantiated the credibility of regenerative approaches that venture on recapitulating neurogenesis and neuronal integration in diseased areas. So far, three distinct strategies for neuronal replacement have been pursued and will be reviewed here in this order: (1) endogenous recruitment from neurogenic niches or local cells (Fig. 1a); (2) transplantation of exogenous cells from neuronal lineage (Fig. 1c); and (3) forced conversion of local glia to a neuronal fate (Fig. 1b). These approaches are at different stages of development, with the first having so far not yet achieved significant and long-lasting neuronal replacement (Fig. 1a, d). Conversely, the second approach has proven to achieve both clinically and experimentally remarkable and meaningful outcome, demonstrating that neuronal replacement is feasible and achieves behavioral recovery in several paradigms (Fig. 1c, d). Finally, the third approach has just now reached sufficient efficiency in vivo to be fully exploited for repair purpose (Fig. 1b, d). Generally, the approaches utilizing endogenous cells for repair purposes (Fig. 1a, b) avoid all issues of good manufacturing practices (GMP) for cells handling and immunological rejection, but are so far less close to clinical application. The approaches using exogenous cells (Fig. 1c) have been successful to unprecedented levels and protocols of manufacturing GMP-grade cells and immunosuppression have been optimized during the last decades. Besides, recent technological advances, such as the discovery of induced pluripotent stem cells (iPSCs) and the emergence of the reprogramming methodology^12^ offer new scalable cell sources that are fit for a precision medicine based on major histocompatibility complex (MHC)-matched cell lines.^13^ Moreover, transplanted cells have been thoroughly probed for their integration into the existing circuitry now to high standards, using sophisticated tools of optogenetic activation/silencing^14–17^ or trans-synaptic tracing to monitor the assembly of their connectome, brain- wide.^18^ Likewise, recovery at the behavioral level has been related to the functional integration of the transplanted cells by silencing them.^17^ These advances now allow a comprehensive understanding of the degree of neuronal integration into operating brain circuits and participation in the respective functional networks. Indeed, it is essential for any neuronal replacement strategy to prove structural and functional repair of the previous circuitry, and provide a causative link with any improvement of behavior. While bystander effects of new cells either recruited or transplanted into the injury site can be very beneficial to improve the clinical outcome, these are not acting as neuronal replacement therapies as they either rescue mature neurons from death and/or promote the above discussed neuronal plasticity. Therefore, as important as these approaches are for the clinics, they do not fall into the subject of our review and the reader is referred to others.^19,20^ In the next sections, we provide an overview of advances and setbacks in the different approaches for neuronal replacement taken so far, present hurdles to act on, and prospects for new clinical trials in patients.

## Endogenous recruitment from neurogenic sources

### Constitutive neurogenesis

In the adult mouse brain, NSCs have so far been detected in the subventricular zone (SVZ) lining the lateral ventricles,^21^ the subgranular zone (SGZ) of the hippocampal dentate gyrus (DG),^22^ and the hypothalamus.^23^ While neurogenesis in the latter is mostly restricted to early postnatal stages, it continues throughout life in the former two regions, albeit much reduced during aging. These two NSC niches appear to be conserved in mammalian species, including humans.^24^ Interestingly, whereas the mouse SVZ produces neuroblasts that migrate substantial distances to reach the olfactory bulb and integrate into the bulbar circuitry largely as granule or periglomerular interneurons, the human SVZ supplies the neighboring striatum with new interneurons.^25^ Bulbar neurogenesis in rodents plays an important role in odor discrimination,^26^ whereas the functional significance of striatal neurogenesis in humans remains to be revealed. On the other hand, the SGZ continuously generates granule neurons of the hippocampal DG and in rodents these are known to be involved in learning and memory, pattern separation,^22^ and perhaps in mood control (debated in Eisch and Petrik^27^). A substantial turnover of dentate granule cells^28^ suggests that adult hippocampal neurogenesis contributes to brain function also in humans and correlative indications implicate reduced neurogenesis in psychiatric disorders. Thus, neurons constantly integrate and replace others in regions of adult neurogenesis (for review, see e.g. Weinandy et al.^29^). This allows studying the mechanisms regulating the synaptic integration of adult-born neurons in pre-existing networks and may help us understanding and fostering neuronal integration in non-neurogenic areas of the adult brain for circuit reconstruction. However, it is important to consider that mechanisms of synaptic competition and selective neuronal survival occur for instance in the DG,^30^ in a fully functional circuit with already optimal neuronal numbers and may hence differ in a setting where neuronal loss leaves gaps in the circuitry and unmatched synapses. Moreover, neuronal integration after injury or in neurodegeneration has to take place in a very different environment, often inflammatory, posing additional challenges for the newly integrating neurons.

### Injury-induced neurogenesis

Brain insults perturb the homeostasis of adult neurogenic niches and the migratory routes of their progeny (Fig. 1a, left). Indeed, the aforementioned rewiring is not the sole attempt of compensation to counteract cell loss, but a spontaneous recruitment of neuroblasts from the neurogenic niches towards pathological sites is observed in a multitude of animal models of brain injury or disease and in *postmortem* brains of patients.

Deviation of SVZ neuroblasts to ectopic sites occurs in response to stroke, trauma, epilepsy and Huntington’s disease (HD) in animal models,^31–37^ as well as in HD and stroke patients^38–41^ (Fig. 1e, left). Importantly, young neurons recruited to the striatum have been reported to differentiate into the appropriate neuronal subtype, namely into DARPP32+ and calbindin+ medium spiny neurons in mouse models of striatal stroke or HD.^31,34,42–44^ This is intriguing, as it implies that injury elicits a profound change in the neuronal subtype produced (normally bulbar interneurons). However, others observed parvalbumin+^45^ or almost exclusively calretinin+^46^ interneurons, or even glia^47,48^ emerging from the SVZ niche to the adjacent injury site, suggesting that these new neurons are less plastic in their neuronal fate choice. When the neuronal subtype is appropriate, to which extent can these recruited neurons connect? They have been shown to form synaptic contacts,^42,43^ receive inhibitory and excitatory input,^49^ but eventually succumb to cell death^31^ (Fig. 1a, d). Indeed, an injury in the striatum is still sensed months later, with large numbers of neuroblasts streaming towards the injury site,^50^ but they fail to achieve long-term replacement of the degenerated neurons.

In addition to the response in the SVZ, stroke and acute epilepsy also foster hippocampal neurogenesis in mouse,^51,52^ while chronic epilepsy^53^ and HD^54^ lead to its decline, but HD patients show no alteration in hippocampal neurogenesis.^55^ In the DG, evidence suggests that increased neurogenesis may be harmful rather than beneficial. Newly generated granule neurons locate also at ectopic positions and extend aberrant projections in the epileptic hippocampus. This may result in additional hyperexcitability of the neuronal network and contribute to epileptogenesis^56–59^ although a causal link is still debated^60–62^ (Fig. 1a, d). In PD and AD, neurogenesis in both neurogenic niches is affected.^63,64^ Progressive dopaminergic denervation of the SVZ in the course of PD leads to impaired neurogenesis, consistent with the well-known role of dopamine in maintaining constitutive proliferation in the SVZ.^65–67^ The DG also receives dopaminergic innervation from the ventral tegmental area and substantia nigra, and 6-OHDA treatment causes a decrease in proliferation of the neurogenic pool and in survival of new granule neurons. However, hippocampal neurogenesis seems to be affected earlier by dysfunctional serotoninergic input originating from the dorsal raphe nucleus due to progressive synucleinopathy.^68–71^ In the case of AD, an impaired neurogenesis both in the SVZ and SGZ was detected before the hallmarks of the disease and may contribute to its progression.^72–75^ However, controversy remains in the case of these two pathologies, as others reported enhanced rather than reduced hippocampal neurogenesis in transgenic PDGF-APPSw mice, a mouse model for AD.^76^ Likewise, neuroblast recruitment to sites of pathology was observed in AD patients^77^ and transient activation of hippocampal neurogenesis has been reported in the 1-methyl-4-phenyl-1,2,3,6-tetrahydropyridine (MPTP) mouse model of PD.^78^

Besides cell mobilization from constitutive neurogenic niches, some studies suggested the generation of neurons from local latent precursors that are activated by an injury (Fig. 1a, right). In the adult neocortex, differentiation into the same projection neuron subtype as previously lost by targeted apoptosis^79,80^ or into inhibitory neurons after cortical ischemia^81^ suggests a local attempt for self-repair. Notwithstanding, neither is the source of these new neurons clear nor are these findings undisputed as later work showed the absence of induced cortical neurogenesis upon targeted apoptosis.^82^

Further evidence shows increased plasticity of glial cells upon injury (Fig. 1a, right). For example, reactive astrocytes isolated from the injured brain parenchyma exhibit NSC-like properties including neurosphere-forming ability and multipotency in vitro, and upregulate some extent of NSC gene expression in vivo.^83–85^ While reactive glial cells are not sufficiently re-specified to generate neurons in most injury conditions in vivo, consistent with the gliogenic nature of this environment,^86,87^ they seem to do so after hypoxia in the postnatal mouse brain,^88^ a possibly more plastic environment. Interestingly, ependymal cells lining the striatum^89^ and striatal astrocytes^43^ can undergo neurogenesis after ischemic stroke due to downregulation of Notch signaling. Accordingly, deleting a central mediator of Notch signaling, RBP-Jk, is also sufficient to allow neurogenesis from these glial cells. While ependyma-derived neuroblasts die early on,^89^ astrocyte-derived neurons seemingly acquire some aspects of medium-sized striatal interneurons, such as nNOS expression, and express synaptic proteins,^43^ while their connectivity and participation in functional circuits still remain to be determined. In line with these findings, Nato et al. used genetic and viral tracing of striatal astrocytes and observed induced neurogenesis upon quinolinic acid treatment, an adult mouse model of HD.^90^ Interestingly, in both models, the injury affects primarily the striatum. So far, a similar transdifferentiation of astrocytes into neurons was not observed in the murine cerebral cortex. However, stroke has been modeled largely in the somatosensory cortex, and curiously only astrocytes in the medial cortex can turn on neuronal traits upon RBP-Jk deletion.^43^ It remains thus open whether brain regions other than the striatum are able to activate endogenous neurogenesis upon injury and if so which signals restrict neurogenesis therein.

Regardless of their source, new neurons are only relevant for cell replacement if they fully mature into the neuronal subtype lost with the pathology, integrate appropriately and survive for long term. Upon targeted apoptosis in the cerebral cortex or in a HD rodent model, injury-induced neurons were able to extend correct long-range projections as shown by retrograde tracer injection in one target area.^44,79,80^ Other than that, it remains rather obscure how injury-induced neurons integrate into brain circuits, which connectome they establish, with no evidence for genuine neuronal replacement and improved circuitry function (Fig. 1d). Furthermore, long-term survival is a major obstacle for the development of strategies that envision cell replacement by boosting this recruitment response. The great majority of the newly recruited neurons dies (0.2% survive at 6 weeks post-stroke) and although the neurogenic response persists for long time,^50,91^ it is not clear if it can be stimulated at a sufficient extent to achieve functionally relevant levels of neuronal replacement associated with a behavioral impact.

These limitations in long-term survival of recruited neurons may be due to a failure to integrate, and/or to cytotoxic effects of the inflammatory environment. Thus, adjuvant treatments further increasing injury-induced migration (e.g. SDF-1alpha, MCP-1, BDNF^92–94^), neuronal specification and survival/proliferation (e.g. BDNF, EPO, SCF, EGF, bFGF, TGFalpha^95–100^) may improve the longer term perspective of this response. A comprehensive knowledge of the effects of the treatment, in the scenario of the disease, is essential. For instance, Fallon et al. describe enhanced neurogenesis and recruitment to injury by TGFalpha infusion in the 6-OHDA rat model of PD, as well as improved apomorphine-induced behavior.^100^ Nonetheless, the authors did not assess neuronal integration and thus alternative effects of TGFalpha, other than neuronal replacement, cannot be excluded. Similarly, bFGF treatment of HD mice leads to increased SVZ neurogenesis and improves motor performance,^44^ but how this is achieved remains ill understood. Although DARPP32+ and BrdU+ neurons were traced retrogradely from dye injections in the globus pallidus, a normal efferent target of striatal medium spiny neurons, the authors also demonstrate a protective role for bFGF in the pathology, implying that motor improvements may result from rescue of pre-existing neurons from cell death. It is paramount to understand if injury-induced neurons can integrate into adult neuronal networks and resume the function of the lost neurons. While endogenous recruitment of neurons still lacks foundations for foreseeing a clinical application, a greater progress has been achieved by use of exogenous neurons or neurogenic cells in transplantation, discussed in the next section.

## Transplantation of neurons or neurogenic cells

While the above approaches are rather restricted to specific brain regions, the introduction of exogenous neurons can be performed in any injured or diseased brain region. Nevertheless, some pathologies are more suitable for this approach than others, and the choice of cell source is deterministic, as discussed below.

First, focal pathologies with mainly one specific neuron subtype lost, such as the degeneration of substantia nigra pars compacta (A9) dopaminergic neurons in PD or striatal medium spiny neurons in HD are best suited for transplantation strategies given the precise brain regions to target and cell type to replace. Stroke and brain trauma are also spatially restricted, but offer greater challenges for neuronal replacement since various types of neurons die within the affected area and the formation of a glial scar is generally thought to be inhibitory to neurite outgrowth.^101^ Yet, it is noteworthy that young transplanted neurons can readily extend axons and seemingly develop well when placed in scar-forming injuries, such as stroke^102^ and cortical aspiration.^103^ The broad and non-cell-type-specific nature of neuronal degeneration in AD render neuronal replacement strategies challenging for this disease. However, transplantation of young inhibitory interneurons, which are highly migratory, surfaces as an option since the interneuron population is dysfunctional in the AD cerebral cortex.^104^ In general, transplantation of migratory cells in multifocal pathologies may benefit from pathotropism, as indeed, homing of NSCs to injury sites relies on a chemoattractant gradient of inflammatory soluble molecules released by the lesioned tissue (reviewed in Martino and Pluchino^19^).

Second, the choice and manufacturing of the source cells is of pivotal importance and advantageous criteria include availability, expandability, easy differentiation into the desired neuron, and MHC-matching with the host. Pioneering work used cells obtained from fetal tissue (ventral midbrain, VM) that are well specified to generate dopaminergic neuronal subtype. Transplantation of these cells into experimental models of PD demonstrated good graft survival and improved motor function thereby launching the field of cell transplantation for brain repair.^105,106^ A series of clinical trials followed in the next two decades, first using autologous adrenal medullary tissue^107^ and then fetal VM tissue^108^ to restore dopamine in the striatum of PD patients, or using fetal ganglionic eminences aiming at cell replacement in HD patients.^109,110^ Fetal tissue grafts showed encouraging results in some PD and HD patients despite variability in the overall patient cohort.^111,112^ For instance, some PD patients developed graft-induced dyskinesias and standardized procedures are now being implemented to reach a more controlled outcome.^111^ Fetal transplants have not only been beneficial in clinical settings, but also shown a long-term survival and a remarkable level and specificity of circuit integration in the adult injured brain, as discussed next.

### Primary fetal neurons

Neurons from fetal sources are superbly specified as they derive from exactly the brain region that generates the neuronal subtype subject to disease. Pioneering studies using ectopic transplantation of fetal midbrain dopaminergic neurons into the striatum of PD animal models or later in patients demonstrated survival and complete maturation into dopaminergic neurons of the correct subtype within the host parenchyma.^111,113^ Newly settled dopaminergic neurons secreted dopamine to the denervated striatum and thus improved behavior. Later on, work by Macklis’ lab paved the way exploring the neuronal integration of fetal projection neurons transplanted into homotopic areas of the adult brain after injury.^114–116^ The team of Gaillard and Jaber published a series of exciting studies that unveiled for the first time how abundantly fetal projection neurons can project through a host parenchyma primed by an injury.^117–119^ Together these studies brought to light a remarkable capability of fetal projection neurons to overcome growth inhibitors of axonal regeneration in the adult brain and project over long distances towards the correct target areas. Interestingly, the above work also highlighted a role for the areal identity of the donor neurons on dictating their projections.^118^ The potential of fetal projection neurons for neuronal replacement therapy received added impetus recently, by demonstration of a close match between the features of the lost neurons and those gradually acquired and tuned in transplanted ones^18^ (Fig. 1c, d). This work showed, for the first time, a comprehensive comparison, brain-wide, of both afferents and efferents of fetal neurons transplanted in the primary visual cortex of adult mouse after an injury. It demonstrated a correct and remarkably precise circuit integration that even re-establishes geniculo-cortical topography. Moreover, the new circuits are functional and tuned in a manner resembling visual cortex neurons, as demonstrated by calcium imaging of transplanted neurons in vivo during visual stimulation.

Transplantation of inhibitory interneurons obtained from fetal ganglionic eminences has also proven successful in reversing excitotoxicity in neurological conditions like epilepsy and chronic pain. Cells from the medial ganglionic eminence (MGE) transplanted into different brain regions develop into mature GABAergic neurons that exhibit identical electrophysiological properties to regular somatostatin and parvalbumin neurons, and enhance local synaptic inhibition.^120–122^ When transplanted into the hippocampus of epileptic mice or into the spinal cord of mice with hypersensitivity after peripheral nerve injury, these cells reduce seizure activity or neuropathic pain, respectively.^123–125^ Moreover, AD as well as traumatic brain injury is often associated with interneuron dysfunction or loss, and consequent imbalance between excitation and inhibition. In AD, this leads to abnormal network activity in the hippocampal DG and memory deficits. Accordingly, transplantation of MGE cells into the hippocampus in an AD rodent model restores normal learning and memory.^104^ MGE cells disperse particularly well from the transplantation site, a feature that may relate with their long migratory routes during development, and which may be deterministic for the success of cell therapy in disorders with widespread neuronal loss as AD. Furthermore, transplantation of fetal interneurons was applied in a few studies in rodents, as a creative strategy to reactivate plasticity at postnatal/adult stages, by reopening critical periods to restore visual perception after early postnatal deprivation^126,127^ or to attenuate recurrence of fear memory.^128^ Interneurons play a central role sculping neuronal circuits activity and plasticity, and by reopening developmentally transient windows of enhanced plasticity in the adult brain they may also contribute to the success of projection neurons integration mentioned above, where donor cell population includes a minority of interneurons. Along the same lines, interneuron transplantation might promote plasticity of the remaining endogenous neurons and improve rewiring or changes in synaptic strengths in the pursuit of functional compensation, discussed previously.

In summary, fetal neurons have provided most exciting results as donor cell population and have been applied in the clinical setting in PD and HD patients^111,112^ (Fig. 1e, right). The achievements made hitherto hold great promise and inspired the raise of the European initiative TRANSEURO, which seeks consistency in the efficacy of fetal cell transplantation in PD patients, and to lift the concern of transplant-induced dyskinesias by careful standardization of criteria for patient selection, cells quality and delivery, and immunosuppressive treatments.^111^ The limited availability of fetal neurons, however, hampers fetal neuron-based cell therapies for neurological disorders and hence efforts have been channeled towards the use of expandable cell sources.

### eNSCs-derived neurons

As expandable cells sources either multipotent NSCs of embryonic origin may be used (eNSCs), or pluripotent stem cells, discussed next. Both of these cells can be expanded efficiently in vitro, but the main challenge is their differentiation into the disease-relevant neuronal subtype. Early transplantation studies from Evan Snyder and colleagues tested the immortalized C17.2 cell line, originally obtained from neonatal mouse cerebellum. These works showed neuronal and glial differentiation after transplantation,^129^ neurite outgrowth,^130^ and a protective role towards the host degenerating neurons.^131^ Besides, C17.2 cells could be differentiated into dopaminergic neurons with high efficiency by Nurr1 overexpression and astrocyte-derived factors.^132^ Concomitantly, the team of Ron McKay isolated and expanded eNSCs from E12 rat VM, using FGF2-mediated neurosphere formation. These cells were subsequently differentiated into dopaminergic neurons by withdrawal of the mitogens, and eventually transplanted in a rat model of PD^133^ leading to a substantial improvement in amphetamine-induced rotation scores. An improved protocol of differentiation was proposed later by Arenas and collaborators including additional patterning factors, namely, FGF8, Shh, and Wnt5a.^134,135^ Interestingly, McKay’s team also isolated expandable eNSCs from cortex or VM of human fetal brain and could generate dopaminergic neurons from both, but only the VM-derived dopaminergic neurons survived in the striatum of parkinsonian rats.^136^ Others also isolated eNSC from human fetal brain tissue, so called human CNS-stem cells^137^ (huCNS-SC) using fluorescence-activated cell sorting (FACS) of CD133+/CD34−/CD45− cells. These cells, highly expandable in vitro and multipotent also after transplantation,^138^ were generated under GMP conditions (StemCells Inc.) and used as donors for multiple approaches in rodent models of SCI, AD, or hippocampal neuronal loss.^139,140^ The research grade huCNS-SC elicited behavioral recovery as assessed in locomotor or cognitive tasks in SCI or AD models, respectively.^139,140^ These findings propelled the translation into clinics and huCNS-SC were transplanted in children with the lethal lysosomal storage disorder neuronal ceroid lipofuscinosis (NCL)^141^ (Fig. 1e, right). This resulted in favorable safety assessments and was followed by transplantations into thoracic SCI patients (unpublished). The mechanism that leads to the observed improvements, however, remains unclear, and seems at least partially due to a bystander effect. Recently, two studies describe efficacy failure of clinical grade huCNS-SC in rodent models of SCI and AD^142,143^ and highlight the importance of testing safety and efficacy for individual clinical grade lots and of performing long-term assessments.

### ESCs-derived and iPSCs-derived neurons

Since the isolation of human ESCs (hESCs)^144^ great efforts were made to design and improve the generation of specific neuronal subtypes relevant for cell replacement therapy and testing their functional integration into the CNS in animal models of disease. Almost a decade later, human somatic cells were reprogrammed for the first time into iPSCs (hiPSCs) with just a handful of genes,^145^ a discovery that set the stage for a new momentum in the brain regeneration field, by offering a scalable and MHC-matched source of neuronal and glial cells from equivalent ground state cells.^13,146^ Substantial progress has been made on optimizing the directed differentiation of pluripotent ESCs or iPSCs toward a given neuronal fate by defined culture settings that activate or inhibit master developmental pathways (for review see Steinbeck and Studer^147^). Alternatively, direct lineage conversion from a somatic cell, like skin fibroblasts, to a neuron of interest (induced neuron, or iN),^14,148,149^ or reprogramming of somatic cells into induced neural progenitor cells (iNPC),^150–152^ which can then be guided toward the desired fate, skip the pluripotent stem cell intermediate and thus carry no risk of tumor formation after transplantation.

Transplantations into the developing rodent brain, an environment that naturally supports neuronal maturation and synaptic integration, demonstrated the therapeutic potential of ESCs-derived and iPSCs-derived neurons.^153–160^ These reports showed survival and integration into the developing host circuits by anterograde/retrograde tracing and/or electrophysiological recordings. Notably, ESCs-derived and iPSCs-derived neurons are also able to survive and extend long-range projections to target areas in the adult injured brain^103,161,162^ and improve behavior in PD animal models.^163,164^ Indeed, VM-patterned hESCs seem to provide functional benefits with similar efficiency to human fetal VM neurons^162^ although innervating less target areas of A9 dopaminergic neurons. Recently, the correlation between gene expression profiles of various VM-patterned hESC lines and their outcome after transplantation into a rodent model of PD identified a set of caudal midbrain markers that predict enhanced dopaminergic neuron yield.^165^ Moreover, single cell transcriptomics of VM Lmx1a progenitors showed that several markers routinely used for dopaminergic lineage patterning are shared with neuronal lineages from subthalamic nuclei, and propose the application of the unique dopaminergic markers to better tailor the source cells for cell replacement in PD.^166^ Altogether, these findings highlight the importance of guiding cells to the very exact neuronal subtype for the best outcome upon transplantation.

Excitingly, optogenetic silencing of grafted hESCs-derived DA neurons proved for the first time a causative link between the graft synaptic transmission and improved behavioral outcome.^17^ Also, hESCs-derived GABAergic projection neurons can be generated with great efficiency and integrate into HD-like degenerating circuits improving motor function.^167^ Notably, hiPSCs-derived cortical neurons survive in an extremely inhospitable environment as stroke-lesioned parenchyma,^16,168^ receive input from correct host brain areas including the thalamus and respond to sensory stimulation.^16^ Those hiPSCs-derived grafts also improved sensorimotor function already 2 months after transplantation.^102^ At this time point, the input connectome was already established with no further change for the next 4 months,^16^ showing a fast formation of afferent connections. On the other hand, most of the grafted cells were still expressing the immature neuronal marker doublecortin raising the question to which extent the behavioral effects were due to circuit integration and/or bystander effects. Indeed, one limitation observed over the years is the rather protracted period of neuronal maturation from human pluripotent stem cells, which constitutes a major bottleneck for their routine and large-scale application in disease modeling or regenerative medicine. This has been facilitated by improved protocols with accelerated neuronal differentiation either using transcription factors^169,170^ or small molecules.^160^ Alternatively, this obstacle can be overcome by using direct conversion of human fibroblasts into the desired neurons.^14,148,149,171–175^

Furthermore, studies to date highlight the need to sort out the remaining pluripotent stem cells to exclude tumor formation upon grafting^176^ or improve the differentiation protocols efficiency to avoid those tumorigenic contaminants and other unwanted neural types. On the other hand, extensive expansion in culture is associated with increased genomic and epigenomic instability,^177,178^ a hallmark of malignant cells. This concern can be tackled by the use of standardized culture settings that minimize genomic alterations. Additionally, stringent preclinical safety tests must assess both purity of the donor cell population and genomic/epigenomic integrity.

In summary, neurons derived from pluripotent cell sources have reached a stage where they become comparable to those derived from fetal brains. Indeed, preclinical assessments of hESCs or hiPSCs as sources for neuronal replacement therapy are encouraging and motivated the large-scale generation of GMP-qualified cell products for clinical use. At present, phase I/II trials have been initiated in patients with age-related macular degeneration and Stargardt macular dystrophy transplanted with hESCs-derived retinal pigment epithelium (RPE)^179,180^ (Fig. 1e, right) and the next years will witness clinical translation also to PD patients (Gforce-PD).^181^ In addition, autologous hiPSCs-derived RPE was transplanted in a patient with age-related macular degeneration.^182^ This study was suspended due to safety concerns but it is prospected to be resumed using allogeneic MHC-matched hiPSCs.

## Direct neuronal reprogramming in vivo

As glial cells are involved in scar formation after acute injury or in maintaining neuroinflammation in chronic neurodegenerative conditions, it would be particularly elegant to turn these cells into neurons. This would allow alleviating the disease conditions and at the same time replace degenerated neurons. As any endogenous replacement approach it avoids the difficulties of clinical grade cell manufacturing, transplantation and concerns of graft immunogenicity and rejection. This approach has been well-established in vitro starting with astrocytes from the postnatal mouse brain that were converted into neurons by transduction with various neurogenic fate determinants.^183,184^ Importantly, cells (pericytes) from the adult human brain^185^ can be converted into neurons in vitro. However, as anticipated, to transfer direct neuronal reprogramming into the complex environment of the brain posits a greater challenge. Initial experiments showed exciting proof of principle evidence that proliferating glial cells reacting to injury can be converted into young neurons in vivo.^186^ Thereafter, the progress stalled for a few years as induced neurons were rather immature, the conversion rate was low, and most neurons died early on. Recently, however, a breakthrough was achieved by blocking both cell death with co-expression of Bcl2 and excessive levels of reactive oxygen species by giving anti-oxidants, such as vitamins D and E.^187^ This is sufficient in combination with a single neurogenic factor, Neurogenin 2, to convert about 90% of infected proliferating glial cells into neurons after stab wound injury (Fig. 1b). Importantly, these neurons also survive longer and develop not only the complex morphology of pyramidal neurons but also acquire traits of neuronal subtypes, namely transcription factors characteristic of deep layer neurons in the cerebral cortex. Interestingly, both proliferating astrocytes and oligodendrocyte progenitor cells (OPCs) of the stab wound-injured mouse neocortex can be converted into glutamatergic deep layer neurons in vivo by forcing NeuroD1 expression, but only the latter cells generate also GABAergic neurons,^188^ indicating a key role of the starting cell. In a model of AD, the authors report increased efficiency of reprogramming from reactive astrocytes in the old as compared to the young AD mouse, suggesting a crucial influence of the environment.

There is also an interesting difference between brain regions with striatal glial cells turning into mature neurons upon Sox2 expression with the additional help of BDNF,^189,190^ while cortical glia-derived neurons remain largely immature when reprogrammed by Sox2 or fail to convert at all if no injury precedes.^191^ In the striatum, both OPCs^192^ and astrocytes^189,190,193,194^ can be reprogrammed into the neuronal lineage. Interestingly, Ascl1/Lmx1a/Nurr1 converts intact striatal OPCs into mainly GABAergic and some glutamatergic, but not dopaminergic neurons.^192^ On the other hand, direct reprogramming of striatal astrocytes into dopaminergic neurons has been achieved recently by priming the environment with an injury and adding a microRNA.^194^ Ascl1/Lmx1a/NeuroD1 together with miR218 convert striatal astrocytes into dopaminergic neurons, which resume dopaminergic transmission and even partially recover functional deficits in a PD mouse model. Given this breakthrough in achieving behavioral improvements by direct neuronal reprogramming it will be important to examine the connections of these neurons. Dopaminergic neurons lost in PD are located in the VM, but transplanting dopaminergic neurons into their target area, the striatum, has been shown to have positive effects as discussed above. However, little is known about the connections that are behaviorally relevant at this ectopic site and it will be fascinating to compare transplanted and reprogrammed neurons in regard to their circuitry. RABV-mediated monosynaptic input tracing has been used to determine the input to reprogrammed neurons in the striatum, namely the GABAergic and glutamatergic neurons mentioned above, but only local input could be detected.^192^

Taken together, these exciting recent data highlight where the field stands at the moment. High efficiency of reprogramming has been achieved in several instances in injury conditions in vivo, and some studies now also achieved further differentiation into specific neuronal subtypes. However, circuitry-relevant aspects remain to be determined in homotopic areas where neuronal loss takes place. Indeed, it is now essential to determine the output projectome of the induced neurons, their brain-wide input connectome and finally test whether a behavioral improvement is due to their participation in the circuitry by silencing them using optogenetics. In other words, the gold standards reached in the transplantation field now also have to be applied here.

In summary, a causal link between synaptic integration of induced neurons in diseased/injured circuitries with an observed functional outcome is central to envision the application of this approach in regenerative medicine. Furthermore, it is compelling to ensure that the deviation of non-neuronal cells to another function does not become detrimental, by understanding at which stage after an injury specific glial cells can be converted into neurons without interfering with the advantageous aspects of glial reactivity, like constraining the spread of the damage. Along these lines, alternative candidates for local reprogramming may be microglial cells and infiltrating macrophages given that germ-line differences imply no major boundary for neuronal reprogramming anymore.

## Concluding remarks

Taken together, the field of neuronal replacement has approached a very exciting state, not only because several approaches are close to clinical application, but also because circuit and optogenetic analyses have demonstrated a high level of specificity when neurons integrate and engage in injured/diseased circuits and participate in behavioral recovery. While these fascinating data have provided the critical proof of concept, the focus on neuronal circuitry and neuronal subtype-specific function highlight the tasks ahead. For example, as transplanted neurons do extend projections to correct target regions, now the next step is to address the speed of this communication. This implies examining not only if the axons of the transplanted or reprogrammed neurons are myelinated, but whether they are myelinated with a profile that allows the correct timing of action potentials.^195,196^ Work of the past decade fueled by technical advances, e.g. on single-cell sequencing has further amplified our knowledge on neuronal diversity, with an increasing number of subtypes and subtype-specific specializations, including processes, such as myelination, that were thought to be rather general. Indeed, the field has successfully addressed the task of generating the disease-relevant neuronal subtypes as best exemplified by the generation of dopaminergic neurons. While initially the quest was to generate ‘dopaminergic neurons’ it became obvious that a specific subtype of dopaminergic neurons (from the ‘A9’ nucleus, or substantia nigra) work best for behavioral recovery after transplantation into animal models. Then the race was on generating this specific type and indeed by combining developmental insights about their region of origin, mimicking the signals in vitro and utilizing the power of single-cell sequencing, it became possible to generate this precise neuronal subtype with high accuracy and efficiency in vitro. This degree of achievements has still to be reached with reprogrammed or recruited neurons in vivo, a challenge that must be pursued in order to develop less invasive strategies for neuronal replacement in highly specialized neuronal networks. Thus, the future in neuronal replacement therapy lies much in the field of neuronal circuits and it will be exciting to see these two fields interacting more closely, not only for the benefit of repair, but probably also to better understand neuronal networks and their functioning.
